# Predictors of Emergency Department Use among Individuals with Current or Previous Experience of Homelessness

**DOI:** 10.3390/ijerph16244965

**Published:** 2019-12-06

**Authors:** Morgane Gabet, Guy Grenier, Zhirong Cao, Marie-Josée Fleury

**Affiliations:** 1 Département de Gestion, Évaluation et Politiques de Santé, École de santé publique, Université de Montréal, 7101 av. du Parc, Montréal, QC H3X1X9, Canada; morgane.gabet@umontreal.ca; 2 Research Center, Douglas MH University Institute, 6875 LaSalle Blvd, Montreal, QC H4H 1R3, Canada; guy.grenier@douglas.mcgill.ca (G.G.); zhirong.cao@douglas.mcgill.ca (Z.C.); 3 Department of Psychiatry, McGill University, 1033, Pine Avenue West, Montreal, QC H3A 1A1, Canada

**Keywords:** predictors, emergency department, homelessness, type of housing, healthcare service use, mental health, substance use disorder, stigma

## Abstract

This study assessed the contributions of predisposing, enabling, and needs factors in predicting emergency department (ED) use among 270 individuals with current or previous experience of homelessness. Participants were recruited from three different types of housing (shelter, temporary housing and permanent housing) in Montreal, Quebec (Canada). They were interviewed at baseline (T0), and again 12 months after recruitment (T1). Longitudinal data analyses were conducted on associations between a set of baseline predictors (T0) with the dependent variable (ED users vs. non-users) from T1. Predictors were identified according to the Gelberg–Andersen Behavioral Model. Findings revealed two needs factors associated with ED use: having a substance use disorder (SUD) and low perceived physical health. Two enabling factors, use of ambulatory specialized services and stigma, were also related to ED use. No predisposing factors were retained in the model, and ED use was not associated with type of housing. Improvements are needed in SUD and physical health management in order to reduce ED use, as well as interventions aimed at stigma prevention for this vulnerable population.

## 1. Introduction

Emergency department (ED) overcrowding and misuse are constant and increasing concerns worldwide [1], not least in Canada and in the province of Québec [2]. ED use in Canada increased 6% between 2005 and 2017 [3], while research identified 2.8 million ED visits in Quebec in 2016–2017, representing a 1.3% rise in ED use compared with 2014–2015 [4]. ED overcrowding has had an important negative impact, as evidenced in increased wait times [5], and reduced patient safety, comfort and satisfaction [6,7]. This has led to concerted efforts among policy makers and healthcare professionals to find ways of reducing ED use [2]. ED overcrowding is partially due to the prevalence of mental disorders (MD) among ED users [8], including substance use disorders (SUD) [9]. Individuals with psychiatric diagnoses, such as schizophrenia [10] and co-occurring MD/SUD [11], are more likely to make high use of ED. Homeless individuals who are particularly affected by MD and/or SUD also account for the high incidence of ED overcrowding [12]. The Gelberg–Andersen Behavioral Model for Vulnerable Populations [13] suggests factors that may predict ED use, among the many variables with specific pertinence for homeless populations. Predisposing factors are individual characteristics (e.g., age, sex), including beliefs and attitudes that may influence ED and other service use. Needs factors involve reported health issues (e.g., user perceptions of physical or mental health) or health status (e.g., diagnoses), while enabling factors are variables that facilitate access to healthcare services (e.g., having a regular source of healthcare) [6].

Most studies concerned with assessing ED use in the homeless population have compared the results with the general population [12,13,14]. According to these studies, homeless individuals were 3–4 times more likely than others to use ED in any given year. Predisposing factors known to increase ED use among homeless individuals, compared with non-homeless individuals, included male gender [14,15], older age [14,16], and housing insecurity [17,18]. Needs factors were most strongly associated with ED use among homeless individuals [19]. Having MD [14,20,21], such as schizophrenia or depression [20] as well as SUD [12,14,16,20,21,22,23], were major factors. Other needs factors related to ED use in this population have included chronic physical illness such as HIV and tuberculosis [20,24], high risk of suicidal behaviors and co-occurring MD/SUD [25]. Enabling factors were also found to influence ED use. Poor social support [20], poor access to social services, and weak patient satisfaction with health services increased ED use among homeless individuals [14]. By contrast, having health insurance [15] and greater access to primary care [19] reduced ED use considerably. However, the association between access to specific services (e.g., family doctor, ambulatory care) and ED use among homeless individuals has been poorly studied [26,27]. 

Few studies have compared homeless ED users with non-ED users [23]. As well, few studies that assessed ED use among homeless individuals used a conceptual framework such as the Gelberg–Andersen Behavioral Model. One important predictor of ED use in the homeless population not often considered is housing status [17]. While recent studies on ED use among homeless individuals have designated “being housed vs. not housed” as a key independent variable [22,28], the further distinction between types of housing (e.g., temporary housing or permanent housing) was not made. Moreover, previous studies have focused on a single type of housing, and the effects of this housing on ED use, with or without a control group (e.g., homelessness). For example, permanent housing programs such as Housing First were found to reduce ED use [29]. A US study reported that individuals living in shelters or in temporary housing had higher rates of ED use than permanently housed individuals [30]. Nonetheless, a recent Canadian study that compared these same housing groups did not find any significant differences in ED use according to housing status [31]. Therefore, a major contribution of the present study resides in the use of a sample that emphasized the representation of different types of housing. 

A better understanding of the variables that predict ED use among individuals with current or previous recent experience of homelessness and living in a variety of different housing types may help tailor interventions to their specific needs and reduce ED visits. Using the Gelberg–Andersen Behavioral Model as a conceptual framework, this study identified predictors of ED use in a sample of 270 currently or previously homeless individuals living in three types of housing in Quebec (shelter, temporary housing and permanent housing), who were followed over a 12 month period after recruitment. In order to address important gaps in the literature, the survey for this study also included variables rarely tested in association with ED use for this population (e.g., stigma, perceived mental health and perceived physical health, use of primary care, and types of housing). Based on the literature and the Gelberg–Andersen model, we hypothesized (1) that ED use among individuals with current or previous experience of homelessness would be mainly associated with needs factors, followed by enabling and predisposing factors and (2) that ED use among individuals with current or previous experience of homeless would be associated with types of housing, favoring permanent housing.

## 2. Materials and Methods 

### 2.1. Study Setting and Data Collection

This longitudinal study included two data collection periods: baseline (T0) and a 12 month follow-up (T1). Participants were recruited in Montreal, the city with the largest homeless population in Quebec (Canada). Participants were recruited from community organizations (*n* = 27) offering services for homeless individuals, including housing resources (*n* = 20) such as emergency shelters (*n* = 5), short- or medium-term temporary housing (*n* = 12), and permanent housing with or without supports (*n* = 3). These organizations, and others without a housing component (*n* = 7), also provided essential services including food banks, day centers, leisure activities, employment counseling, and financial or material support. 

In order to participate in the study, individuals had to be at least 18 years old, currently homeless (i.e., using shelters or temporary housing), or have previous experience of homelessness (i.e., living in permanent housing with or without supports for less than two years prior to study recruitment). Participation was voluntary. Interviews for participants identified as intoxicated or otherwise indisposed at the time of recruitment were postponed. Potential participants were informed of the study through posters displayed in the selected organizations, postcards, or by organization staff or research team members (coordinator or interviewers) directly; presentations were also given in the organizations. A total of 497 individuals were invited to participate in the study: 46 emergency shelter users, 243 temporary housing residents, and 208 permanent housing residents. Study participants also agreed to be interviewed a second time at 12 months following recruitment (T1). They provided the research team with their phone numbers, addresses, emails, and the names of reference persons who could be contacted by interviewers between T0 and T1 as well as places that they frequented regularly. Study participants also authorized the team to communicate with the Quebec welfare agency should they not be located using the contact information provided. Telephone contact was the preferred strategy, followed by e-mail and postal mail. Individuals were contacted for a follow-up interview (T1) between one and 17 times, with an average of 3 attempts before they were reached. 

The T0 data were collected between January and September 2017, and T1 data between January and December 2018. The instruments used for data collection are described in Table 1. Interviews at T0 and T1 were conducted by trained research assistants at the selected organizations, participant apartments, or in quiet corners of local cafés or fast food restaurants. Quality of the interviews was closely monitored by the coordinator and research team. Average interview length was 75 minutes for T0, and 55 minutes for T1, as the T1 questions referred only to participant conditions in the previous 12 months. All participants had to sign a consent form before completing the interview and were assured that their responses would remain confidential. They received a modest financial compensation for their time and contribution to the study. The research ethics board of a mental health university institute approved the multisite study protocol.

### 2.2. Variables and Instruments

The dependent variable was ED users (vs. non-users) for individuals with current or previous experience of homelessness. ED visit was assessed using the Service Use Questionnaire [37]. Based on the Gelberg–Andersen Behavioral Model for Vulnerable Populations [13], predictors included the following needs factors: suicidal behaviors (ideation and attempt), perceived physical health, perceived mental health, depressive disorder, post-traumatic stress disorder, anxiety disorders, bipolar disorders, psychotic disorders, personality disorders, SUD (no distinction made between substances), chronic physical illness, and co-occurring disorders. Predisposing factors encompassed age, sex, education, marital status, employment, chronic homelessness (i.e., being homeless for a period of least 12 months, or 4 times within a 3 year period [38]), and housing status. Enabling factors included has a family physician, has a case manager, consulted a psychiatrist in previous 12 months, public primary care use in previous 12 months, community primary care use in previous 12 months, ambulatory specialized service use in previous 12 months, overall satisfaction score, number of social supports used, stigma score, and quality of life score.

### 2.3. Analysis

Missing values (less than 5%) were randomly distributed and treated using the simple imputation method [39]. Longitudinal data analyses were conducted for associations between baseline predictors (T0) and the dependent variable (ED users vs. non-users) from T1. Based on the testing of differences in the effect between groups (ED users vs. non-users), the study would have adequate power (>80% at the 5% significance level) on an estimate of 123 per group [40]. Stata version 15 was used for bivariate and multivariate analyses. The reference category for the logistic regression model was non-users. Odds ratios were calculated for each independent variable. Significant independent variables (with Alpha set at 0.10) in the bivariate analyses were entered sequentially into the multivariate analyses. The alpha value was set at 0.10 to make the analysis less restrictive, because some covariates identified as not significant in bivariate analyses, with the alpha value at 0.05, could become significant when tested against other variables in the multiple regression analyses. The Akaike Information Criterion (AIC) was used for selection of a set of multiple regression models, and the model with the smallest AIC was chosen for the adjusted analysis. Odds ratios were calculated with 95% confidence intervals for multiple regression analyses. 

## 3. Results

Of *n* = 497 recruited, 455 participants were enrolled at baseline (T0), for an overall response rate of 92%. Of the 455 participants, 270 (59%) were interviewed at T1. Comparative analyses using cross tabulation on categorical variables showed no differences in sex between the T0 and T1 samples (*p* = 0.518). T-Tests were used for the continuous variables, age and disability, at T0 and T1, yielding no significant differences for either variable (age: *p* = 0.126, disability: *p* = 0.677). As well, no significant differences were found between individuals lost to follow-up (*n* = 185) and those remaining in the study (*n* = 270), in terms of baseline characteristics for sex (*p* = 0.199), education (*p* = 0.689), and disability score (*p* = 0.330).

Sample characteristics are presented in Table 2. Of the 270 participants, 51% were ED users (*n* = 137). Regarding predisposing factors, 42% of the sample was female, 57% were 50 years old and older, and 94% were single. Concerning housing status, 50% of participants resided in temporary housing, 44% in permanent housing, while 6% were currently homeless and using shelters. Slightly more than half of participants (52%) were chronic homelessness. In terms of needs factors, 38% of participants reported depression, 19% anxiety, 69% personality disorders, 37% SUD and 35% chronic physical illness. The mean perceived physical health score was 3.33 on a 5-point scale, or “fair”, with a similar result for the perceived mental health score (3.72/5). In terms of enabling factors, 57% of participants had a family physician, and 50% a case manager. Most participants had used primary care services in the previous 12 months (74% public service, 85% community services), and 30% had used specialized ambulatory services. The mean score for overall satisfaction with services was 4.0/5, or “good”. The average score on quality of life was “good” (71/100), and the stigma score was 6.5/8, or “good“.

Variables significantly associated with ED use in the bivariate analysis (Table 2) were selected to build the multiple regression model (Table 3). No predisposing factors were significantly associated with this model. Regarding needs factors, SUD (odd ratio (OR): 1.70, confident interval (CI): 1.01–2.87) were associated with ED users, while patients with better perceived physical health were less likely to be ED users (OR: 0.75, CI: 0.58–0.98). Regarding enabling factors, individuals with higher stigma scores (i.e., less perceived stigma) were less likely to be ED users (OR: 0.70, CI: 0.56–0.89). Finally, greater use of specialized ambulatory services also predicted ED use (OR: 1.74, CI: 1.00–3.01).

## 4. Discussion

This study examined predictors of ED use among 270 individuals with current or previous experience of homelessness. In our sample, half of participants (51%) used ED during the 12 months between baseline and T1, as compared with 33% to 40% in previous studies [17,25,41,42,43]. The greater prevalence of chronically homeless individuals (52%) in our sample might explain the higher proportion of ED users in this study. Chronic homelessness in the US has been estimated at 10% to 15%. Individuals experiencing chronic homelessness are often affected by multiple chronic health conditions, making them more prone to high ED use [42]. Moreover, the proportions of individuals using public primary care (74%) or community services (85%) were also very high, which may be explained by the universal healthcare system in Quebec/Canada, as well as the plethora of community organizations in Montreal providing social services for this population. Compared with findings related to service use in countries without public healthcare, this finding coincides more closely with the results for other countries with universal healthcare, where homeless populations tend to use health services at higher rates [44]. Nevertheless, only a minority had a family physician or a case manager, pointing to the need for better access to these professionals.

The results confirmed the first hypothesis that needs factors would be most strongly associated with ED use, followed by enabling factors. Two specific needs factors (SUD and poor perceived physical health) were positively associated with ED use, confirming their influence on the homeless population [7,19,25]. The strongest predictor was a diagnosed SUD, which is very prevalent among individuals with current or previous experience of homelessness [12,14]. Individuals with SUD were also identified as among the highest ED users [22]. SUD has been directly linked to increased physical health conditions (e.g., intoxication, trauma, infections) [45] and mental health problems [25]. Not surprisingly, homeless individuals with SUD were also reported to be frequent ED users [21,22,25]. Moreover, homeless individuals with SUD reported poor quality of life, and may have experienced housing instability, compromising access to healthcare and leading them to seek care at the ED [18,22,44]. Many patients with SUD, especially those with co-occurring MD, also figure prominently among those exhibiting life-threatening behaviors such as suicidal ideation or attempts, which are usually highly associated with ED use [25,46]. Finally, SUD may have a negative effect on medication and treatment effectiveness [47], which may lead in turn to frequent ED use. Another condition potentially linked to SUD, perceived physical health, was the second strongest needs factor predicting ED use in the study model. User perceptions of physical health, especially in vulnerable populations such as the homeless, are crucial in evaluating the adequacy of services used [19], and, to the best of our knowledge, have not been previously tested. Even in the general population, lower perceived physical health is directly associated with ED use [48]. A recent cohort study of individuals affected by MD found that ED visits were mainly related to physical health conditions [48]. Moreover, people with MD and/or SUD are known to have a disproportionate incidence of physical health issues, with significant chronicity [48]. Therefore, perceptions of health closely approximate the actual health conditions of individuals, leading to increased ED visits [24]. Overall, chronic physical illness or co-occurring MD/SUD affected more than a third of the study sample, suggesting an increased need for and greater use of health services. However, these variables as well as the presence of other MD such as anxiety or suicidal behaviors (significant in bivariate analyses) were not significant in the final model where all the variables were considered and, as such, did not significantly distinguish ED users from non-users in this homeless sample. This finding is contrary to the results of previous research that compared the homeless population to the general population [14,20,21]. The fact that no particular MD were associated with ED use in this study may be explained by the overall high prevalence of MD in the study sample, especially personality disorders.

Two enabling factors were significant predictors of ED use among homeless or previously homeless individuals (specialized ambulatory service use, and stigma score). The association between specialized ambulatory service use and ED use is not surprising, as this clientele is known to utilize specialized services for the most part [41,44]. However, very few tests of “ambulatory” specialized service use in association with ED use have been conducted in previous studies with homeless populations [49]. Those making the greatest use of ED may be the most vulnerable in terms of multiple health and social problems, which is what likely drove them to make high use of specialized ambulatory services as well. Previous studies show that frequent ED users are also high users of services in general [25]. Yet the intensity of specialized ambulatory services, or care continuity among the various services, may have been inadequate, explaining why ED were a more viable alternative for homeless individuals in difficult moments [24]. Moreover, very little integrated treatment is available in Quebec, which implies that users affected by co-occurring MD/SUD, including most homeless individuals, may be redirected from one health organization to another, eventually driving them to ED [50]. Concerning stigma, the present study is one of very few that have integrated this variable into a model explaining ED use among homeless individuals. What is known is that homeless individuals are often victims of stigmatization, not only from the general public but also from clinical professionals [51]. Due to past negative experiences with health services, some homeless individuals may refuse to seek help, even at ED. Homeless individuals are also more likely to arrive at the ED by ambulance or with a police escort, as compared with other ED users [15,52]. In previous studies, stigma was also found to be higher among homeless individuals with MD or SUD, or those with criminal records, suggesting a double burden on the former [53]. In addition, homeless individuals often suffer from internalized stigma, which is associated with lower self-esteem, lower levels of hope, and more depressive symptoms [54], leading them to ED [53]. Not least, homeless individuals are often victims of violence which may lead them to seek treatment at ED [55].

No predisposing factors were significant in the model. Studies on ED use or high ED use among individuals with MD or SUD produced inconsistent results in relation to age [56,57,58]. While male gender was usually associated with ED use in such previous studies [14,15], no difference was found between male and female participants in this study sample. As well, poor socioeconomic conditions were usually found to increase ED use [51], yet this finding was not confirmed in the present study, probably because all participants, including those living in permanent housing, were unemployed and receiving very little social assistance.

The results did not confirm our second hypothesis that ED use among individuals with current or previous experience of homelessness would be associated with type of housing. This result is surprising, as previous studies have found that permanent housing, particularly in combination with case manager support, may contribute to decreased ED use, and fewer hospitalizations, among homeless individuals [29,31,59,60,61,62]. One explanation may be that some of the main factors affecting ED use, such as SUD, low perceived physical health and stigma, were prevalent in our sample, regardless of participant housing status. It may also be that the implementation of permanent housing in Quebec has occurred too recently or perhaps inadequately to show an influence on ED use [63,64]. Case management was especially underused by study participants, even among permanent housing residents. Another explanation might be that individuals using shelters, the group mostly frequently compared with permanent housing residents, were underrepresented in our sample.

This study has some limitations that should be addressed. First, our convenience sample may not represent the entire Quebec homeless population, especially in terms of housing status, age or study areas. Individuals using shelters, and youth, were particularly underrepresented. Second, as our sample was quite heterogeneous, including individuals living in both permanent and temporary housing, as well as those using shelters, the results cannot be generalized to any specific type of housing. Third, participation in the study was voluntary, suggesting that our findings would require further validation. Fourth, the study data did not provide information on the particular types of substances used by homeless individuals, which may have produced different patterns of ED use. Fifth, the results of this study reflect the nature of the Canadian health system and its features (e.g., universal coverage), which might preclude generalization to other healthcare systems without such extensive coverage. Finally, a study period longer than 12 months may have allowed for the identification of further predictors of ED use.

## 5. Conclusions

This study was among very few that have analyzed ED use among homeless or previously homeless individuals based on a conceptual framework. Several innovative variables, such as stigma and perceived physical health, and related to enabling factors, mostly services variables, were included. The results showed that having SUD, low perceived physical health, low perceived stigmatization, and greater use of specialized ambulatory services predicted ED use among homeless individuals, regardless of housing status. This suggests that public initiatives to prevent homelessness should focus on SUD prevention and treatment. Shared care initiatives, such as the integration of addiction liaison nurses in ED, might improve screening for SUD, referral and treatment continuity in outpatient services. Better collaboration between SUD treatment and health and social services with ED may also reduce frequent ED use for this clientele. Moreover, greater access to family physicians and case managers, and improvements in the overall responsiveness of primary and ambulatory services, may offer better care alternatives than ED use to individuals, particularly those with more negative perceptions of their physical health. Finally, the results of this study underline the necessity for public authorities and healthcare professionals to initiate actions promoting stigma prevention and more targeted care for this vulnerable population, even in services such as ED.

## Figures and Tables

**Table 1:** Survey instruments measuring clinical and service use variables.

Name	Variables	Description	Psychometric Properties
Service Utilization Questionnaire (SUQ)	Professionals and services used in previous 12 months, and satisfaction with services	Five-point scale (1 to 5)	N/A
M.I.N.I International Neuropsychiatric Interview 6.0 [32]	Mental disorders (MD) 120 items	Two-point scale (no = 0; yes = 1)	Kappa Cohen = 0.50–0.84
Standardized Assessment of Personality Abbreviated Scale [33]	Personality disorders 8 items	Two-point scale (no = 0; yes = 1)	Cronbach’s alpha = 0.68
Alcohol Use Disorders Identification Test (AUDIT) [34]	Alcohol abuse 10 items	2 multiple-choice questions rating: 0–50; higher = greater level of substance use disorder	Cronbach’s alpha = 0.74
Drug Abuse Screening Test (DAST) [35]	Drug abuse 20 items	Two-point scale (no = 0; yes = 1) rating: 0–20; higher = greater drug use disorder	Cronbach’s alpha = 0.88
Satisfaction with Life Domains Scale (SLDS) [36]	Subjective quality of life 20 items	Five-point scale (1 to 5) rating: 20 to 100; higher = better quality of life	Cronbach’s alpha = 0.92

**Table 2:** Characteristics of study participants according to emergency department (ED) use and bivariate analyses.

Factors	Total (*n* = 270)		Non-Users ED (*n* = 133)		ED Users (*n* = 137)		Bivariate Analyses	
	*n*/ Mean	%/ SD	*n*/ Mean	%/ SD	*n*/ Mean	%/ SD	OR	*p*-Value
Predisposing factors (T0)								
Age: 18–39 years	14	5.19	5	3.76	9	6.57	-	-
40–49 years	103	38.15	54	40.60	49	35.77	0.50	0.247
50 and over	153	56.67	74	55.64	79	57.66	0.59	0.368
Sex: female	114	42.22	58	43.61	56	40.88	0.89	0.649
Education (college or over)	88	32.59	46	34.59	42	30.66	0.84	0.491
Marital status: in couple	12	4.44	5	3.76	7	5.11	1.38	0.592
Employed	21	7.78	10	7.52	11	8.03	1.07	0.876
Chronic homelessness	141	52.22	72	54.14	69	50.36	0.86	0.535
Housing: shelter	17	6.30	5	3.76	12	8.76	-	-
Temporary housing	134	49.63	67	50.38	67	48.91	0.42	0.118
Permanent housing	119	44.07	61	45.86	58	42.34	0.40	0.100
Needs factors (T0)								
Suicidal behaviors (ideation and attempt)	64	23.70	23	17.29	41	29.93	2.04	0.016
Perceived physical health (mean/SD) ^1^	3.33	0.99	3.48	0.98	3.18	0.97	0.73	0.015
Perceived mental health (mean/SD) ^1^	3.72	0.88	3.89	0.75	3.55	0.96	0.63	0.002
Depressive disorder	102	37.78	43	32.33	59	43.07	1.58	0.070
Post-traumatic stress disorder	14	5.19	5	3.76	9	6.57	1.80	0.304
Anxiety disorders	51	18.89	17	12.78	34	24.82	2.25	0.013
Bipolar disorders	37	13.70	14	10.53	23	16.79	1.72	0.138
Psychotic disorders	59	21.85	26	19.55	33	24.09	1.31	0.368
Personality disorders	187	69.26	88	66.17	99	72.26	1.33	0.278
Substance use disorders (SUD)	101	37.41	40	30.08	61	44.53	1.87	0.015
Chronic physical illness (CPI)	94	34.81	40	30.08	54	39.42	1.51	0.108
Co-occurring disorders: mental disorders (MD/SUD)	94	34.81	37	27.82	57	41.61	1.85	0.018
MD/CPI	85	31.48	36	27.07	49	35.77	1.50	0.125
SUD/CPI	46	17.04	15	11.28	31	22.63	2.30	0.015
MD/SUD/CPI	43	15.93	14	10.53	29	21.17	2.28	0.019
Number of CPI (mean/SD)	0.6	0.98	0.46	0.77	0.74	1.13	1.36	0.022
Enabling factors (T0)								
Has a family physician	153	56.67	71	53.38	82	59.85	1.30	0.284
Has a case manager	136	50.37	67	50.38	69	50.36	1.00	0.999
Consulted a psychiatrist in previous 12 months	84	31.11	32	24.06	52	37.96	1.93	0.014
Public primary care use in previous 12 months ^2^	199	73.7	94	70.68	105	76.64	1.36	0.266
Community primary care use in previous 12 Months ^3^	229	84.81	116	87.22	113	82.48	0.69	0.280
Specialized ambulatory service use in previous 12 months ^4^	81	30.00	31	9.77	50	36.50	1.89	0.019
Overall satisfaction with services score (mean/SD)	3.99	0.76	4.10	23.31	3.88	0.77	0.68	0.021
Number of social supports used (mean/SD) ^5^	2.18	2.44	2.25	2.37	2.12	2.51	0.98	0.658
Stigma score ^6^ (mean/SD)	6.53	1.15	6.76	1.00	6.31	1.24	0.69	0.001
Quality of life score (mean/SD) ^7^	71.05	9.76	72.64	9.61	69.0	9.69	0.97	0.009

^1^ The higher the score, the better the perception of physical health or mental health. ^2^ Service use includes family doctors or other general practitioners in walk-in medical clinics at local community health service centers (LCHSC) or at private clinics, and other LCHSC services (e.g., psychosocial services). ^3^ Addiction treatment centers, support groups, women’s centers, employment support programs, or other community organizations (e.g., day centers, food banks). ^4^ Hospital services other than hospitalization or ED, and addiction rehabilitation center services. ^5^ Participants were asked to identify the number of significant persons they could confide in or relate to in difficult moments. ^6^ Scores ranging from 2 to 8: higher scores indicate less perceived stigma. ^7^ Quality of life score, range from 20 to 100: higher scores indicate better quality of life. SD—standard deviation, OR—odd ratio.

**Table 3:** Determinants of emergency department (ED) use according to the multiple logistic regression model.

Determinants	OR	*p*-Value	95% CI Low	95% CI High
Needs factors (T0)				
Substance use disorders (SUD)	1.70	0.047	1.01	2.87
Perceived physical health	0.75	0.032	0.58	0.98
Enabling factors (T0)				
Specialized ambulatory service use ^1^	1.74	0.049	1.00	3.01
Total stigma score ^2^	0.70	0.003	0.56	0.89
Constant	18.70	0.001	3.10	112.92

^1^ Hospital services other than hospitalization and ED, and addiction rehabilitation center services. ^2^ Scores range from 2 to 8: higher scores indicating less perceived stigma. OR—odd ratio, CI—confident interval.
